# Association between HIV/AIDS and Multi-Drug Resistance Tuberculosis: A Systematic Review and Meta-Analysis

**DOI:** 10.1371/journal.pone.0082235

**Published:** 2014-01-08

**Authors:** Yonatan Moges Mesfin, Damen Hailemariam, Sibhatu Biadglign, Kelemu Tilahun Kibret

**Affiliations:** 1 Department of Public Health, College of Medical and Health Science, Haramaya University, Harar, Ethiopia; 2 School of Public Health, Addis Ababa University, Addis Ababa, Ethiopia; 3 Freelance Public Health Research Consultant, Addis Ababa, Ethiopia; 4 Department of Public Health, College of Medical and Health Science, Wollega University, Nekemte, Ethiopia; University of Illinois at Chicago, United States of America

## Abstract

**Background:**

Human immunodeficiency virus (HIV), multi-drug resistant tuberculosis (MDR) is emerging as major challenge facing tuberculosis control programs worldwide particularly in Asia and Africa. Findings from different studies on associations of HIV co-infection and drug resistance among patients with TB have been contradictory (discordant). Some institution based studies found strongly increased risks for multi-drug resistant TB (MDR TB) among patients co-infected with TB and HIV, whereas other studies found no increased risk (it remains less clear in community based studies. The aim was to conduct a systematic review and meta-analysis of the association between multi-drug resistant tuberculosis and HIV infection.

**Methods and findings:**

Systematic review of the published literature of observational studies was conducted. Original studies were identified using databases of Medline/Pubmed, Google Scholar and HINARI. The descriptions of original studies were made using frequency and forest plot. Publication bias was assessed using Funnel plot graphically and Egger weighted and Begg rank regression tests statistically. Heterogeneity across studies was checked using Cochrane Q test statistic and I^2^. Pool risk estimates of MDR-TB and sub-grouping analysis were computed to analyze associations with HIV. Random effects of the meta-analysis of all 24 observational studies showed that HIV is associated with a marginal increased risk of multi-drug resistant tuberculosis (estimated Pooled OR 1.24; 95%, 1.04–1.43). Subgroup analyses showed that effect estimates were higher (Pooled OR 2.28; 95%, 1.52–3.04) for primary multi-drug resistance tuberculosis and moderate association between HIV/AIDS and MDR-TB among population based studies and no significant association in institution settings.

**Conclusions:**

This study demonstrated that there is association between MDR-TB and HIV. Capacity for diagnosis of MDR-TB and initiating and scale up of antiretroviral treatment, and collaborations between HIV and TB control programs need to be considered and strengthened.

## Introduction

Tuberculosis (TB) is a chronic infectious disease mainly caused by *mycobacterium tuberculosis* (MTB). Occasionally caused by other organisms of the Mycobacterium tuberculosis complex- *M. bovis*, *M. africanum*, *M. canetti* and rarely, *M. microti* [1].

TB has been causing great suffering to human beings throughout recorded history. Even two decades after introduction of DOTS control strategy, tuberculosis still remains a major cause of morbidity and mortality worldwide [2]. One-third of the world's population is estimated to be infected with mycobacterium tuberculosis [2]. In 2010, there were 8.8 million (8.5–9.2 million) incident cases of TB, 1.1 million (0.9–1.2 million) deaths from TB among HIV-negative people and other 0.35 million (0.32–0.39 million) deaths from HIV-associated TB. There were also 1.4 million deaths from TB in 2010 and among this 1.1million were HIV-negative people and an additional 0.35 million deaths from HIV-associated TB [3]. HIV, MDR-TB, and XDR-TB are jeopardizing the TB control program worldwide [4].

World Health Organization (WHO) has documented that MDR-TB is emerging as a major challenge for tuberculosis control programs and is becoming extensively widespread today throughout the world, even in high-income countries with low TB incidence. It is a challenge not only from a public health point of view but also in the context of global economy, especially in the absence of treatment for MDR-TB at national programs level in developing countries [4].

According to data in 2010, about 650 000 cases of MDR-TB, which account for 5% of all newly diagnosed TB patients, and more than 150,000 MDR-TB deaths are estimated to occur worldwide each year with case fatality rate of 30 per 100 individuals [4]. The proportion of MDR-TB reported globally ranges from 0% to 28.3% and 0% to 61.6% among new TB cases and among previously treated TB cases respectively [5]. The magnitude of MDR-TB is not known precisely because of the lack of prevalence information from all countries. Most sub–Saharan African countries have been unable to carry out the necessary laboratory investigations because of the absence of proper equipment to identify the M. tuberculosis strains resistant to the two drugs used in the first -line treatment [6].

People living with HIV are at a higher risk of developing MDR and XDR tuberculosis associated with increased mortality, and greatly reduced survival time [7]. HIV and MDR-TB are equally balanced deadlier combinations [8]. Even if the impact of HIV infection on MDR-TB is of great public health importance, the relationship between the two infections is not yet clearly understood. Findings from different studies on associations of HIV co-infection and drug resistance among patients with TB have been contradictory (discordant). Some institution based studies found strongly increased risks for multi-drug resistant TB (MDR TB) among patients co-infected with TB and HIV [9]–[12], whereas other studies found no increased risk (it remains less clear in community based studies) [13]–[15]. The question of whether HIV co-infection places a person at increased risk for drug resistance remains largely unanswered.

This study summarized the evidence of the association between HIV infection and multi-drug resistance tuberculosis through a systemic review and meta-analysis of studies

## Methods

### Study design and data source

Systematic review and Meta analysis of the published literature of observational studies was conducted. Original studies providing Mycobacterium tuberculosis resistance data stratified by HIV status were identified through a computerized search using databases of Medline/Pubmed, Google Scholar and HINARI (Health Inter Network Access to Research Initiative) with detailed search-strategy and cross-checking of reference lists. The search terms used to search the database were drug resistance, multidrug resistance, MDR-TB, HIV/AIDS and any of the following—risk factors for MDR-TB, epidemiologic determinants for MDR-TB, clinical determinants for MDR-TB, predictors of MDR-TB, correlates of MDR-TB, surveillance on MDR-TB, and surveys on MDR-TB - were used as a combination of free text and thesaurus terms in different variations. The International Journal of Tuberculosis and Lung Disease was selected as the key journal for hand searching. Search was also made for cross-reference lists of identified original articles and reviews for other relevant articles. The data abstraction was performed from October 1 to April 10, 2012.

### Study Selection

A systematic review and Meta analysis was made on observational studies (cross-sectional, surveillance/survey, case-control and cohort studies) which were reported on the association of HIV infection and MDR-TB. Eligibility criteria for articles to be included in the meta-analysis were if they presented results based on drug susceptibility to rifampicin and isoniazid of mycobacterium tuberculosis and stratified by HIV status (independent of study design and without restriction of publication date). Reports of original studies, unpublished master's thesis and PhD dissertations which were written in English language also considered while comments, editorials and reviews were excluded. Studies were excluded from the analysis for any of the following reasons: articles focused only on extra-pulmonary tuberculosis; those dealing with a mycobacterium other than tuberculosis; those that did not consider HIV as risk factors (independent variable); studies that do not give effect estimates in odds ratios, rate ratios, or risk ratios, or did not allow the computation of such; studies among children <15 years; meta-analyses or systematic reviews; duplicate publication of the same study; and articles available only in abstract form, articles with sample size of less than 50 were also excluded. The selection of articles for review was done in three stages: titles alone, abstracts, and then full-text articles.

### Methodological quality assessment

Method of confirmation of TB, MDR-TB and HIV status, sample size, describing MDR-TB by HIV status, use of right statistical measurement to assess the association between MDR-TB and HIV infection and assessment and adjustment of potential confounders (demographic, socio-economic and previous TB treatment and known contact with TB patient related variables) were noted as quality of indicators. Reporting of response rate, lost to follow up and appraisal of external validity of study result were also considered as study quality indicators.

All assessments were entered into pre-formatted standardized data extraction forms. Studies were assessed for quality and studies with medium (fulfilling 50% of quality assessment parameter) and high quality were included for analysis. High quality studies were: studies that reported outcomes on at least 50 patients; cohort studies with lost follow up of less than 20%, case control studies (matched or unmatched), cross-sectional studies and surveillances whose response rate was greater than 80%; those that reported basic demographic data, clear stratification for unknown and HIV negative individuals and adjustment for covariates like demographic, socio-economic and previous TB treatment and known contact with TB patient related variables.

### Data abstraction

The data abstraction was conducted independently by two of the investigators (YH, DH). The selected studies were reviewed by using pretested and standardized abstraction form to extract data about title; authors, year of publication, country, study design, study site, study base (population-based or hospital-based), sample size, data collection procedure, TB form (pulmonary, extra pulmonary), type of MDR-TB (primary, acquired TB), HIV status, adjustment and stratification factors, response rates, measure of association like OR/RR with its confidence interval (CI), P-values, proportion of exposed and who developed the disease for different categories. When there was a discrepancy in data abstraction, it was resolved through consensus among the team of investigators.

### Data Synthesis

In accordance with the World Health Organization's definitions for tuberculosis control (36) [16], any drug resistance is defined as resistance to one or more first-line drugs. ‘Mono resistance’ is defined as resistance to only one of the five first-line drugs (INH, RMP, STM, EMB, and PZA). MDR-TB is defined as M. tuberculosis strains that are resistant to at least INH and RMP. Primary or initial resistance, (resistance among new cases), is defined as patients with TB resistant to one or more anti-TB drugs, but who had never been previously treated for TB or had treatment less than one month. ‘Secondary resistance’ (resistance among previously treated cases) is defined as patients diagnosed with TB who started anti-TB treatment and subsequently acquired resistance to one or more of the drugs used during the treatment.

### Statistical Analysis

Epi-info version 3.5.1 and STATA version 11.0 using metan command (STATA Corporation, College Station, Texas) software were used for data entry and analysis respectively. The descriptions of original studies were assessed by using frequency and forest plot. The overall effect (pooled estimated effect size) of HIV infection on MDR-TB was carried out by using the Der Simonian-Laird random-effects meta-analysis (random effects model) and measured by odds ratio with 95% confidence intervals [95%CI].

#### Sub-group analyses

Sub-group analyses were performed according to study base (population based or hospital based), study design (cross-sectional, surveillance/survey, cohort and case-control), type of multi- drug resistance tuberculosis (primary or secondary), adjustment for potential confounders (demographic, socio-economic, previous tuberculosis treatment and known contact) and based on selection of controls (hospital and discharge record based vs population based) for case-control studies.

### Statistical heterogeneity and Exploration of Publication bias

Publication bias was assessed using Funnel plot by displaying individual study OR with 95% confidence intervals (CIs). The Begg rank correlation and Egger weighted regression test methods were also used to statistically assess publication bias (P<0.05 was consider as indicative of statistically significant publication bias). Cumulative meta-analysis also used to see the effect of each study and less precise studies on the pooled estimates. Statistical heterogeneity was assessed with Cochran's Q test, which tests if the amount of between study heterogeneity is greater than due to chance [17] and I^2^ statistic the magnitude of statistical heterogeneity that can be expected by partitioning out the chance heterogeneity [18].

The I^2^ statistic is a measure of the proportion of variability (inconsistency) between studies that is due to chance as opposed to the actual difference between study populations. So, the presence of statistical heterogeneity was tested using Cochran's Q test (P<0.10 considered indicative of statistically significant heterogeneity) and recently developed measures of magnitude of statistical heterogeneity between trials using I^2^ (values of 25%, 50% and 75% are considered to represent low, medium and high heterogeneity respectively).

### Ethical issues

Ethical clearance was obtained from institutional review board (IRB) of school of public health, Addis Ababa University

## Results

**Figure 1:**
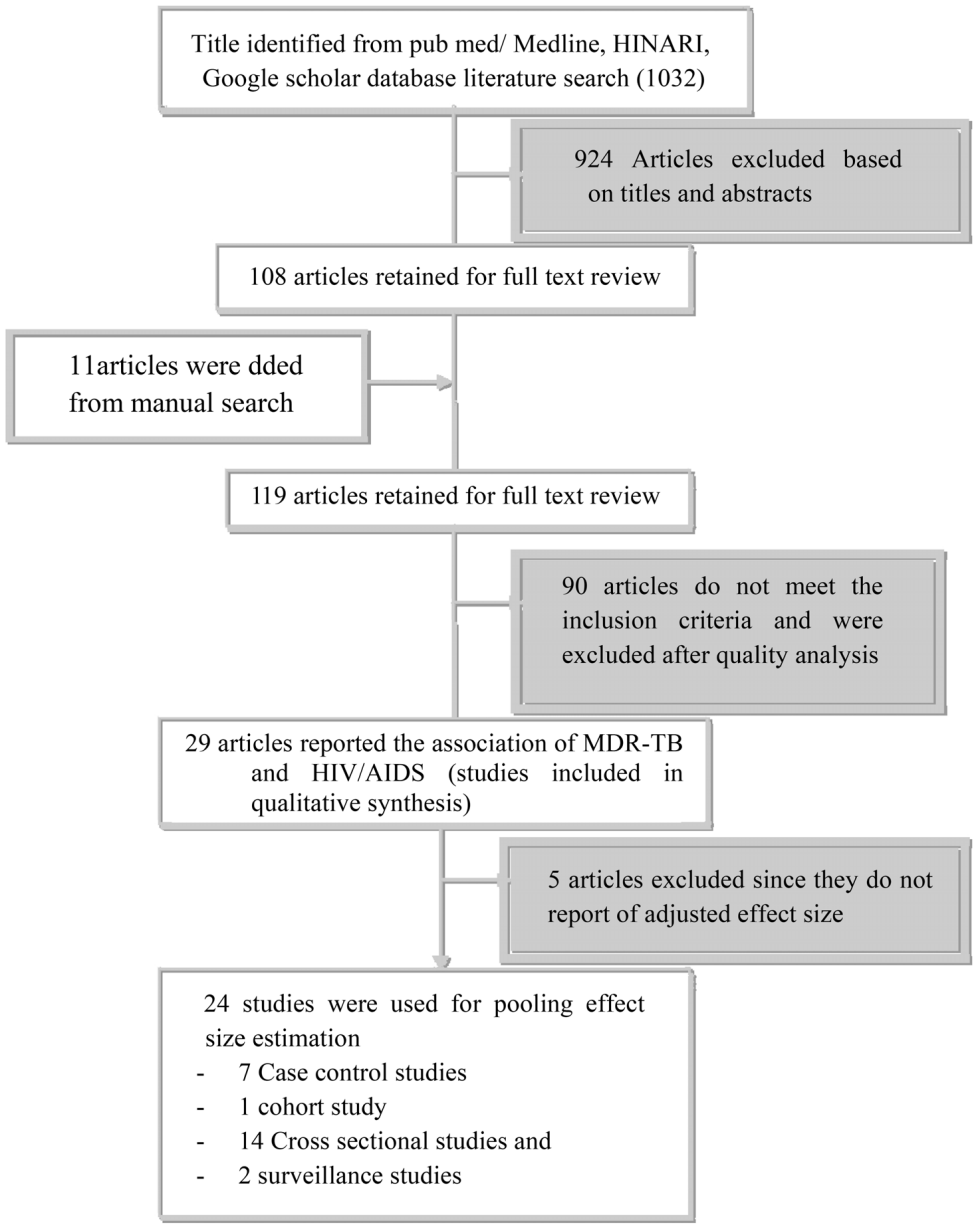
Flow chart diagram describing selection of studies for a systematic review (identification, screening, eligible and included studies). Articles may have been excluded for more than one reason.

A total of 1032 original articles were identified from the initial PubMed, HINARI and Google Scholar search on MDR-TB risk factors and 11 other papers were identified from a manual search of International Journal of Tuberculosis and Lung Disease. Of these, 924 were excluded after screened by titles and abstracts. Those were duplicated studies, and those that were case reports, reviews, or studies of mono drug resistance tuberculosis and extensive drug resistance tuberculosis. Of the remaining 119 articles, 90 studies were excluded because they were studies of MDR-TB other than M. tuberculosis [19]; studies of MDR-TB treatment outcome [20]; they were systemic review and meta-analysis [3]; they did not consider HIV/AIDS as independent variable [21], and access could not be gained to the full article or data (8) [22]; were on children [5]; were dealt only on extra pulmonary tuberculosis [23]; their sample size were less than 50 [23], and did not give a quantitative effect estimate [22]. Further exclusion of studies that did not adjust for covariates [19] was also made. Finally 24 articles were used for the meta-analysis with a total population of 92,714. See **Figure 1** for the flow of the diagram for study selection.

### Characteristics of Studies Included in the review

Seven of the 24 studies selected for meta-analysis were case control studies [17], [18], [24]–[28], one study was prospective cohort study [29] and the rest 16 were cross sectional studies and surveillance [7], [10], [30]–[42]. Twenty two of them were institution based [10], [17], [24]–[28], [30]–[35], [37]–[43] or based on discharge records and two were population based [18], [36]. The resulting 24 studies addressing the association of HIV and MDR-TB, which had study populations varying from 172 in New York City [40] to 55,571 in France [31], were carried out between 1990 and 2011. All studies were reported in English.

The 24 retrieved studies represented 16 countries in 6 regions (South/Southeast Asia, Sub- Saharan Africa, Western Europe, Eastern Europe, Latin America, and North America). General characteristics and description of the studies selected for meta-analysis are outlined in **Table 1**.

**Table 1:** Summary of the 24 observational studies assessing the association between HIV/AIDS and multi-drug resistance tuberculosis included in the meta-analysis.

First author, year, country	Design	Sample size	MDR-type	Number HIV+	MDR-TB HIV+	Number HIV−	MDR-TB HIV−	AOR	CI
Dubrovina. et al, 2008 (Ucrane)	Cross-sectiona^a^	1540	Any	307	31.6%	1143	23.8%	1.7	1.3–2.3
Valerieschoeel et al, 1998 (France)	Case-control^a^	1334	Primary	893	1.2%	5864	0.3%	3.3	1.5–7.3
		4	secondary	107	11.2%	868	6.6%	1	0.5–2.0
Robert M.Granchi. et al, 2005 (California)	Cross- sectional^a^	2871	Any	2031	4%	2736	1.4%		0.78–1.23
		2				2		0.98	
Patrice Josephe. et al, 2006 (Haiti)	Cross- sectiona^a^	330	primary	115	10%	166	3%	3.2	1.1–8.9
Andrew C.weltman. et al, 1994(newyork city)	Case- contro^a^	172	any	78	26.9%	25	4%	2.7	1.1–6.8
Nino Mdivani. et al, 2008 (Georgia)	cross-sectiona^a^	996	any	5	40%	227	28.6%	1.4*	0.47–4.17
Catharina Hendrika. tal,2007(Netherlands)	Cross- sectional^a^	7090	primary	308	1.6%	646	0.6%	2.78	1.09–7.1
Kliiman K. 2009 (Estonia)	Cross sectional^a^	1163	any	54	16.7%	914	18.8%	1.57	0.80–3.11
S.J conaty. et al,2004 (England Wales)	Case - control^b^	9541	Primary	274	3.6%	7936	1%	2.5	1.2–5.20
			secondary	19	21.4%	611	8.2%	2.8	0.6–11.9
k.weyer. et al, 2007 (s/Africa)	Cross- sectional^b^	5866	any	2700	3.4%	1939	2.9%	1.3	1.0–1.70
			Secondary	501	7.9%	418	5.7%	1.46	1.04–2.07

N.B:

^a^ represents institution based studies and.

^b^ represents population studies.

represents unknown HIV status patients or individuals considered as HIV negative.

### Assessing Heterogeneity and publication bias among the studies

**Figure 2:**
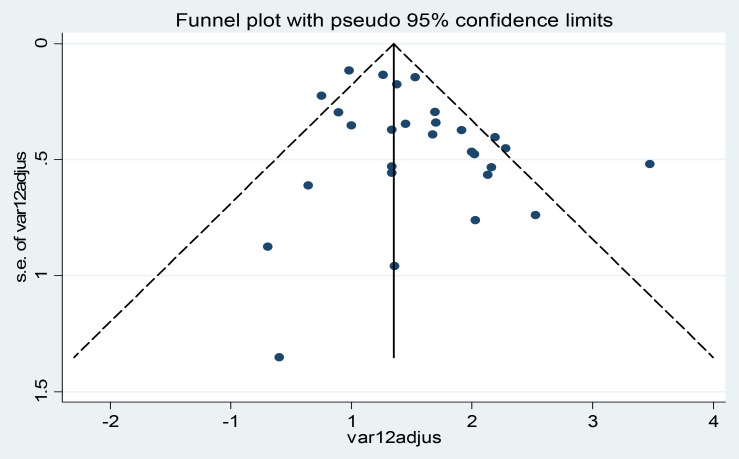
Funnel plot of with 95% confidence limit; the horizontal line in the funnel plot indicates the effect estimate, while the sloping lines indicate the expected 95% confidence intervals.

The studies showed good homogeneity using Cochrane Q test statistic (Q test p = 0.181) but low heterogeneity was observed up to 19.4% (I^2^ = 19.4%) which was indicative for using random- effects model. The distribution of the studies using traditional funnel plot **(** **Figure 2** **)** showed symmetrical distribution of effect estimate and Beg rank correlation statistic (p = 0.33) showed no evidence of publication bias. But Egger weighted regression analysis (p = 0.02) showed presence of publication bias.

**Figure 3:**
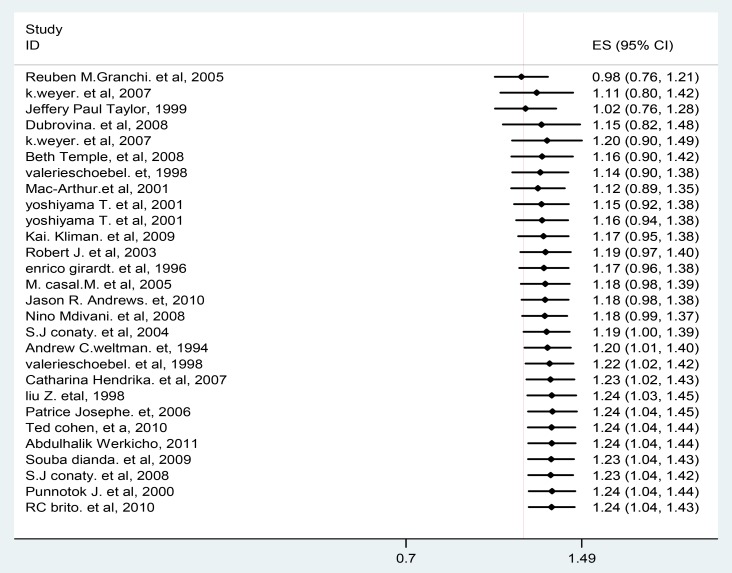
Cumulative forest plot. The first row shows the effect based on one study, the second row shows the cumulative effect based on two studies, and so on.

According to publication bias in meta-analysis – Prevention, Assessment and Adjustments [44], if there is publication bias, the recommendation is displaying the data using cumulative forest plot (the studies sorted from the most precise to the least precise) and need to see the effect of less precise studies (having large effect estimate) on the pooled effect estimate. So, in this meta-analysis if we limited the analysis to the larger studies, the relative risk would have been 1.21 (1.01, 1.40) and if we excluded the last two studies (Punnotok J. et al, 200 and RC.Brito et al, 2010) [33], [34], the publication bias test (Egger weighted regression analysis) was 0.06 and the pooled effect estimate was 1.23 (1.04, 1.43). The clinical implications and even the statistical significance probably would have been the same with the pooled estimate, 1.24 including the last two studies mentioned above **(** **Figure 3** **)**. But there is still need to be interpreting the finding of this study carefully.

### The Association of HIV/AIDS and Multi-drug resistant Tuberculosis

**Figure 4:**
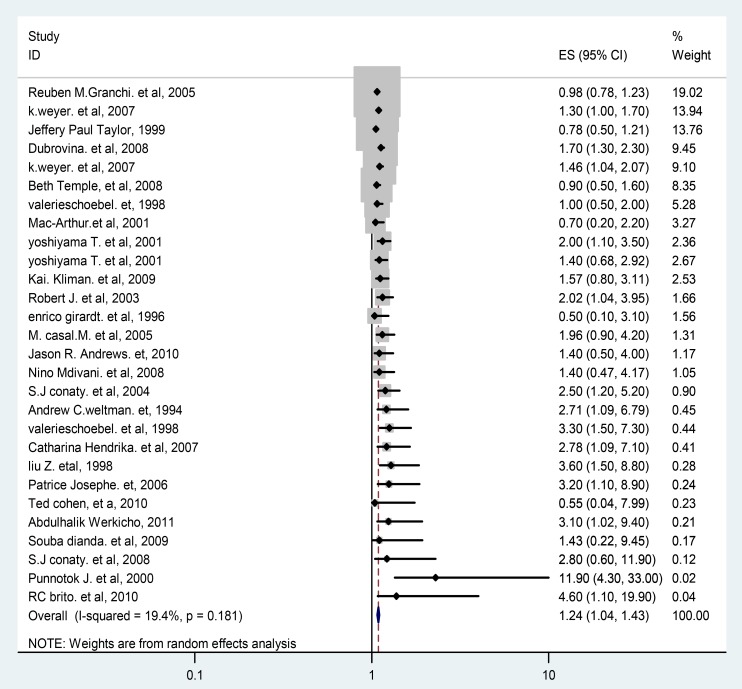
Forest Plot of the 24 observational Studies That Quantitatively Assessed the Association between HIV/AIDS and multi-drug resistance tuberculosis.

Based on the 24 observational studies included in this meta-analysis, the pooled odd ratio according to random effect DL model was 1.24 (95% CI 1.04 to 1.43) **(** **Figure 4** **)**. In this plot, the studies have been listed from most precise to least precise, so that larger studies appear toward the top and smaller studies appear toward the bottom. This has no impact on the analysis, but allows us to get an initial sense of the relationship between sample size and effect size.

Based on sub group analysis; we found moderate heterogeneity of effect estimate from studies within each study design (between study variance accounted for 33% of the total variance among cross sectional studies and surveillance) and low heterogeneity of effect estimate (I^2^ = 19.6%) of the total variance among institution based studies. The pooled effect estimate for primary MDR- TB and HIV (7 studies) was 2.28 (95% CI 1.52, 3.04). The pooled effect estimate for population based studies (2 studies) was 1.38 (95% CI 1.09, 1.66). The sub group analysis output based on study characteristics and quality assessment criteria were summarized in **Table 2**.

**Table 2:** Quality assessment and sub group analysis; HIV/AIDS and multi-drug resistance tuberculosis.

Measure or Outcome	Study Characteristics (Number of Studies)	Summary OR	95% CI	I^2^
**Outcome**	Primary MDR-TB (7)	2.28	1.52–3.04	0%
	Secondary MDR-TB (9)	1.02	0.80–1.24	17.9%
	Any (12)	1.18	1.14–1.64	0%
**Type of study**	Case-control (9) and cohort studies (1)	1.20	0.79–1.58	0%
	Cross sectional and Surveillance (18)	1.26	1.02–1.49	33%
**Study base**	Population (4)	1.38	1.09–1.66	0%
	Institution (24)	1.20	0.96–1.43	19.6%
**Adjustment for**	Demographic and tuberculosis related variables (10)	1.51	1.02–1.99	0%
	Demographic and socio-economic variables (8)	1.33	0.7–1.96	0%
	Demographic,Tuberculosis and socio-economic related variables (8)	1.23	0.96–1.56	54.3%
**Selection of control**	from hospital and discharge records (7)	1.39	0.80–1.99	0%
	From population (2 study)	2.53	0.65–4.42	0%

I^2^ = percentage of total variance due to between- study heterogeneity.

OR = odd ratio of summary estimate.

**Figure 5:**
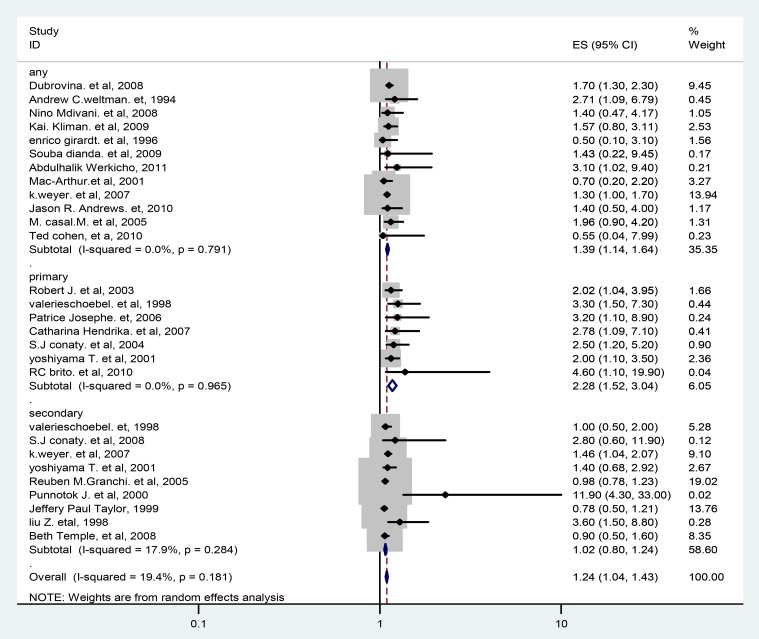
Forest Plot of the 24 Studies That Quantitatively Assessed the Association between HIV/AIDS and multi-drug resistance tuberculosis by MDR-TB type.

The measure of association on HIV/AIDS and MDR-TB by multi-drug resistance type based on the data presented from 24 studies was summarized in **Figure 5**. The plot demonstrated that there is significant positive association between HIV/AIDS and primary MDR-TB with summery OR of 2.28 (95% CI, 1.52–3.04) and there is positive association between HIV/AIDS and secondary (acquired) MDR-TB but not significant (summery OR = 1.02, 95% CI; 0.80, 1.24). There is no heterogeneity at all (Q test, p = 0.791 and I^2^ = 0%) for any type MDR-TB and (Q test, p = 0.965 and I^2^ = 0.0%) for primary MDR-TB.

## Discussion

This systematic review and meta-analysis addressed the association of HIV infection and multi- drug resistance tuberculosis using 24 selected studies. According to the results of this meta-analysis, the odds of having MDR-TB among HIV positive cases was higher by 24% and this was statistically significant as pooled OR of 1.24 (95%, 1.04–1.43) regardless of study designs, study base, and type of multi-drug resistance tuberculosis. But about 81.4% of the weight of the pooled odds ratio of this meta-analysis was comes from cross sectional and surveillance studies which have low power to assess predictors. The subgroup analysis further showed that HIV infection was a risk factor for multi- drug resistance tuberculosis among population based studies than among institution based studies. The pooled risk of primary multi-drug resistance tuberculosis was two times higher in HIV infected population as compared with counterpart not infected with high homogeneity (I^2^ = 0%) between studies.

There was publication bias despite of several studies observed no relationship between MDR- TB and HIV infection included in this review. The cumulative meta-analysis also showed that the increased risk in the larger studies was 21% and 23% for all studies but Punnotok J. et al, 200 and RC.Brito et al, 2010. There was an evidence of bias in pooled estimate based on all studies, and the risk was probably somewhat higher than reported in highly precise studies, but there was no reason to doubt the validity of the core finding, that HIV/AIDS was associated with clinically and statistically significant increase in the risk of multi-drug resistance tuberculosis.

This meta-analysis, unlike previous reviews [45], includes ten recent studies that had examined the association of HIV infection and MDR-TB as well as generating the overall pooled summary estimate regarding the overall association between HIV infection and multi-drug resistance tuberculosis. Additionally a rigorous sensitivity analyses was done to identify important sources of heterogeneity. Unlike previous meta-analysis [45] which didn't report an overall association between MDR-TB and HIV due to a high heterogeneity across the studies. But their subgroup analysis results suggest that HIV infection was associated with primary MDR-TB and not statistically associated with acquired MDR-TB and HIV. The sub group analysis result (for primary and secondary MDR-TB) of the present meta-analysis is consistent with the above systemic review in that HIV infection is significantly associated with primary multi-drug resistance tuberculosis and positively associated but not significant with secondary MDR-TB.

This could be explained by the fact that HIV infected patients have a rapid disease progression and in settings where MDR-TB is prevalent, either in the general population or in the local population such as a hospital or a prison. This may lead to rapid development of a pool of drug resistant TB patients, or an outbreak. Furthermore, people living with HIV may also be more likely to be exposed to MDR-TB patients, due to either to increased hospitalizations in settings with poor infection control or association with peers who may have MDR-TB, including in prison settings [5].

Several biological mechanisms also linking drug resistant TB to HIV infection documented. People with HIV infection progress from tuberculosis infection to active disease faster than immune competent people. Drug mal-absorption in HIV infected patients, especially rifampicin and ethambutol, can lead to drug resistance and has been shown to lead to treatment failure [26].

Consistence finding has been observed [46] on risk factors for multi-drug resistance tuberculosis in Europe that MDR-TB patients were more likely to be HIV positive.

Surveillance of anti-tuberculosis drug resistance in the world: an updated analysis, from 2007–2010 (surveillance data from 17 countries and 1 territory were combined) [47] reported that the odds of having MDR-TB among HIV-positive cases were found to be 40% higher than among HIV negative but the difference was not statistically significant. The possible explanation for the discrepancy of the findings could be explained by methodological difference of the studies included in the meta-analysis and the study was based on patients' visiting health institutions. Around 18.4% of the weight of the pooled odd ratio of our meta-analysis comes from case-control studies and cohort studies that have chosen with strong design.

The finding of this analysis should be interpreted in the context of both inherent limitation of the original studies and the current review and meta-analysis.

There are several potential limitations to this study. The analysis was based on estimates derived from observational studies that are vulnerable to confounding by variables associated with both HIV infection and MDR-TB. To address the issue of potential confounding, a sensitivity analysis was performed in which separate summary estimates was reported for the studies that adjusted for important potential confounders and those that did not. Studies that controlled for socioeconomic status in a multivariable model found that the adjusted effect of HIV infection was reduced. Although it is not possible to exclude the possibility of residual confounding by unmeasured confounders in these observational studies, such as other chronic diseases that often coexist with HIV, the effect of HIV infection on MDR-TB risk was found to persist even after adjustment for multiple potential confounders that are likely to be correlated with unmeasured factors.

Seven of the case control studies included in this meta-analysis used different approaches to the selection of controls, including sampling from hospitals, discharge records, department of health records and the general population. Sampling controls from hospital or discharge records may have introduced a Berkson bias. Other potential sources of bias include possibly misclassification of both exposure and outcome status.

## Conclusion

In summary, this study found consistent but marginal evidence for an increase risk of MDR-TB among people with HIV infection despite some heterogeneity was observed among cross sectional, surveillance studies and institution based studies. Sub-group analysis also showed that HIV infection was positively and significantly associated with multi-drug resistance tuberculosis among population based studies and also with primary multi- drug resistance.

The results have programmatic implications and public health importance. Capacity for diagnosis of MDR-TB and initiating and scale up of antiretroviral treatment, and collaborations between HIV and TB control programs need to be considered and strengthened. Ensuring early case detection, diagnosis through quality-assured bacteriology and provide standardized treatment with supervision and patient support, Good infection control program need to be implemented and there also need to have close follow up to reduce risk of spread of MDR-TB, especially in HIV positive patients, particularly in clinics and hospital set up.

## Supporting Information

Checklist S1 PRISMA Checklist.
(DOC)
