# Correction: Association between HIV/AIDS and Multi-Drug Resistance Tuberculosis: A Systematic Review and Meta-Analysis

**DOI:** 10.1371/journal.pone.0089709

**Published:** 2014-02-26

**Authors:** The PLOS ONE Staff

The name of the fourth author is spelled incorrectly. The correct name is: Sibhatu Biadgilign. The correct Citation is: Mesfin YM, Hailemariam D, Biadgilign S, Kibret KT (2014) Association between HIV/AIDS and Multi-Drug Resistance Tuberculosis: A Systematic Review and Meta-Analysis. PLoS ONE 9(1): e82235. doi:10.1371/journal.pone.0082235
